# The feasibility of smartphone-based application on cardiac rehabilitation for Chinese patients with percutaneous coronary intervention in Macau: a qualitative evaluation

**DOI:** 10.1080/17482631.2021.2023940

**Published:** 2022-01-17

**Authors:** Sarah Sio Wa Lao, Sek Ying Chair

**Affiliations:** a Department of Nursing, Kiang Wu Hospital, Macau SAR, China; b Graduate Division, the Nethersole School of Nursing, Faculty of Medicine, The Chinese University of Hong Kong, Hong Kong SAR, China

**Keywords:** mhealth, cardiac rehabilitation, qualitative evaluation, percutaneous coronary intervention, Chinese patients

## Abstract

**Background:**

To improve cardiovascular risk factors modification and effects of cardiac rehabilitation (CR), electronic applications on CR are suggested in the literature for patients after percutaneous coronary intervention (PCI).

**Methods:**

A sequential qualitative study, embedded in a quantitative experimental trial for mHealth application on CR (mCR) study evaluation, was conducted to understand the usability and satisfaction of mCR study. Purposive sampling were used until achieving data saturation. Individually semi-structured interviews were conducted. The textual narration from interview transcriptions were analysed by content analysis.

**Results:**

Ten participants were interviewed for qualitative evaluation. Findings presented the perceptive and experience of the mCR app users. Results were captured by four themes: 1. feasibility of mCR app, including practicality, acceptability and convenience, and barriers to use; 2. benefits from mCR app, explaining the effectiveness of mCR study; 3. advocator for better hospital care, disclosing an extension of healthcare and promoting patient-healthcarer relationship; and 4. recommendation for mCR app improvement.

**Conclusion:**

Findings provided insights for cardiac healthcare providers to understand the feasibility of mHealth application on phase II CR in Macau. The mCR app facilitated CR engagement which contributed to health and well-being by promoting CHD and CR knowledge, and cardiac healthy lifestyle modification.

## Introduction

### Coronary heart disease prevalence

Cardiovascular disease (CVD) is the number one underlying cause of global death, nearly 17.7 million deaths every year and 31% of all deaths worldwide (World Health Organization [WHO], 2018). Coronary heart disease (CHD) has become the major leading cause of death (42.6%) in CVD since 2017 (American Heart Association [AHA], 2017, 2020). Heart disease in Hong Kong is the third leading cause of death and CHD is the dominant component holding 66.6% of heart disease mortality according to the data from Hong Kong Department of Health in 2019 (Hong Kong Department of Health, 2019, 2021). Likewise, heart disease is the second leading cause of death in Macau in 2019, and CHD mortality accounts for 75% of all heart disease deaths (Macau Health Bureau, 2017; Macau Statistics and Census Service, 2020). Acute myocardial infarction (MI) has been one of the top 10 major causes of death since 2012. Ischaemic heart disease was the sixth leading causes of death in 2016 (Macau Health Bureau, 2013, 2017).

### Benefits of cardiac rehabilitation after percutaneous coronary intervention

Percutaneous coronary intervention (PCI), with coronary stenting, has become one of the most frequently implemented therapeutic procedures to improve coronary blood flow and to restore myocardial reperfusion by local mechanical or pharmacotherapy treatment (European Society of Cardiology [ESC], 2012). Cardiac rehabilitation (CR), is the Level A-Class I recommendation for patients with stable angina, previous MI, after PCI or coronary artery bypass grafting (CABG) (American Association of Cardiovascular and Pulmonary Rehabilitation/American College of Cardiology Foundation/American Heart Association [AACVPR/ACCF/AHA], 2010; American College of Cardiology Foundation/American Heart Association/Society for Cardiovascular Angiography and Interventions [ACCF/AHA], 2011; ESC, 2012; Woodruffe et al., 2015).

Benefits of CR after PCI are recognized by reducing risks of re-infarction (odds ratio [OR]: 0.53, 95% CI: 0.38–0.76), cardiac mortality (OR: 0.64, 95% CI: 0.46–0.88), and all-cause mortality (OR: 0.74, 95% CI: 0.58–0.95; Lawler et al., 2011). In addition, three systematic reviews and a Cochrane review indicated that there are significant improvements on quality of life (QoL), exercise capacity, smoking cessation, and modification of risk factors, including lipid profile, body mass index, blood pressure (BP) and heart rate after attending CR (Jolly et al., 2006; Lawler et al., 2011; Shepherd & While, 2012; Taylor et al., 2010).

### Alternative CR application for PCI patients in Macau

The application of eHealth/mHealth in CR has been recommended to reduce the risk of recurrent events and to improve self-care management, QoL, medical therapy adherence, and lifestyle modification (Beatty et al., 2013). eHealth is defined as an electronic communication and health information technology in healthcare mHealth is a component of eHealth specifically used in mobile phones, monitoring devices, personal digital assistants, and other wireless devices (Beatty et al., 2013; WHO, 2011). In Macau, there is a difficult service gap to promote CR transition from hospital to home because there is no community centre capable of supporting the phase II CR. Yet, it remains a challenge for the cardiac clinicians to consider how to deliver hospital-initiative CR through a seamless connection between phase I and phase II as to maintain and to maximize the effect and continuity of CR. In addition, the accessibility and convenience of mobile or smartphone-based CR were shown to be more effective than the internet-based CR design (Beatty et al., 2013).

Therefore, a 3-month smartphone-based mHealth application on CR (mCR) randomized controlled trial (RCT) was conducted from November 2017 to April 2019 to support the recovery of Chinese post PCI patients in a non-governmental charitable hospital in Macau. A supplementary qualitative evaluation on the feasibility of mCR study was embedded. Social Cognitive Theory (Bandura, 2004) was adopted in the RCT and the mCR app was hypothesized as the environmental factors to facilitate CR participation. Patients, who were: 1) adult patients aged ≥18 years diagnosed with CHD; 2) received PCI as treatment; 3) eligible to initiate phase I CR during hospitalization and continue phase II CR in OPD CR rehabilitation centre and 4) owned a smartphone, were enrolled in the mCR study. All the target study participants were recruited for random assignment to either experimental group (EG, the mCR group), or control group (CG, the CR booklet group). The use of mCR app for the EG and the CR booklet for the CG were educated after group allocation. All study participants continued to use their assigned CR aids (mCR app for the EG and the CR booklet for the CG) across the study period. Study interventions were delivered by two cardiac Advanced Practice Nurses (APNs). Participants in the EG were registered in the mCR app into their smartphones by the APNs at the first week after hospital discharge in outpatient department (OPD) nurse-led clinic. The participants received a user login identity number and password for mCR app registration, user guide, and education on mCR app utility. A cloud hosting for mCR app data storage was rented to ensure data confidentiality. The mCR app supported four major features, namely, education, health data entry with push-up notification, health status tracking, and interactive communication. The mCR app contained: 1). “Home page”: a personal profile for all mCR users to see their progress and performance throughout CR program; 2). “Educational page”: providing all CR teaching information, including CHD knowledge, self-care skills, physical exercise recommendation, CHD dietary principle and relaxation advices, delivered through video, text, photo and pictures; 3). “Record page”: showing the individualized CR tasks in a checklist format as to remind patients’ engagement in CR recommendations; 4). “Result page”: displaying the CR tasks completion situation and health data input summary; and 5). “Q&A page”: a chatroom to facilitate more interactions and communication between patients and nurses.

## Material and methods

### Study design

Qualitative evaluation was embedded as effectively in helping elucidate the reality underlying mCR app users’ perceived mCR app application experiences in their phase II CR after PCI. Therefore, a descriptive qualitative approach using semi-structured interviews was within the mCR study as to collect information regarding the usability of the mCR app application.

### Settings and participants

Ten mCR app users from EG, who were willing to participate in the qualitative evaluation after program completion within 3 months, were recruited by purposive sampling until data saturation was achieved.

### Data collection

To evaluate the mCR app feasibility, qualitative interview was adopted to collect additional in-depth information. Individual semi-structured and face-to-face interviews, guided by an interview guide (Table I), were conducted. The interview guide was based on literature review (Noh & Lee, 2016) and contained eight broad open-ended questions to elicit narrations of the mCR app user’s experiences. To reach the aims of feasibility evaluation, direct questioning sequence was used for the interview. After a new area was opened, some follow-up questions were asked to support and encourage the interviewee’s descriptions and confirmations (Dahlberg et al., 2008). Reflective and confirmative communication skills were used in the interview to minimize directive guidance of the interviewer.

**Table I.** Interview guide

Interview Guide
Would you please tell me how do you feel about the mCR app program? Relevance Acceptability, convenience Level of difficulty Level of practicality Overall impression More specifically, is there any difference in your **health status** after you participated in the mCR app program? If yes, in what way you feel about the difference? Does such difference impact on your performance/ function in everyday life? Would you talk about any difference in your **health and well-being** after you joined the program? Why there was such difference. Do you agree the mCR app program can **extent the hospital services** in helping patients to follow therapeutic advices in cardiac health? How do you feel about **your experience**? Would you please talk about your home practice? To certain extent, do you enjoy the program? Why? Have you continued with the mCR app practice after the end of program? If yes, what is your experience? Why? What do you want the hospital or government (healthcare system) to support this program in a long run? Do you have any recommendations? Please give me your comments.

### Data analysis

Data collection and data analysis were performed simultaneously. After each interview, the textual narration from the interview transcriptions was analysed using content analysis. Krippendorff (2004) defined content analysis as a research technique for marking replicable and valid inference from text or other meaningful matter to the context of their use. To manage the qualitative data, the repeated listening of audio-recorded interviews and transcription verbatim in Chinese were required after each interview. Accuracy was obtained by rechecking the Chinese transcripts with the audio-recording and the backward translation was undertaken by the second author to ensure the equivalence of the translated materials. A list of remarkable statements about the mCR app users’ experiences and perceptions were identified and coded. Each new code was compared with the previously developed ones, which later became subcategories. Each code, subcategory, and category were carefully prepared to avoid repetitiveness and overlap. Finally, the categories were refined, and the essences of feasibility in mCR app application were presented (Burns, 1997; Creswell, 2007; Streubert Speziale & Carpenter, 2011).

### Rigour

According to Lincoln and Guba’s framework, the trustworthiness of the study finding was assured through four criteria to judge the rigour of qualitative research, including credibility, dependability, confirmability and transferability (Krefting, 1991; Morse & Field, 1995; Polit & Beck, 2008; Streubert Speziale & Carpenter, 2011). Interviews were made consistent by using an interview guide to assure credibility. The schedule contained eight questions and expansions of these questions. Credibility was obtained by inviting the participants to confirm the finding to be true to their experiences immediately after data analysis following each interview and conducting discussion with research supervisor about the research process. Dependability was obtained by showing the description of research methods, including sampling, data collection, and data analysis, which provided information on the repeatability of the qualitative evaluation. The use of methodological expert and research supervisor to check whether the research findings were consistent if the inquiry was replicated was another strategy to ensure dependability. To ensuring confirmability, we recorded the documents throughout the qualitative evaluation, such as field notes of interviews, audio recording, research proposal, notes of research process, instrument of data collection, and records of data analysis. Such records provided an audit trail for another individual to trace. Thus, confirmability was achieved when the degree to which the findings solely from the informants was determined. Transferability was achieved when nominated sampling was adopted. The background data of each participant, such as age, educational level, and the utility of mCR app, were provided as an information or research context to allow others to judge the transferability of the study. Research findings were discussed with the nursing staff in the cardiac ward, CR team members, and research supervisor to check whether the finding could be transferred and whether it was analogous to other discipline (Krefting, 1991; Morse & Field, 1995; Polit & Beck, 2008; Streubert Speziale & Carpenter, 2011).

### Ethical considerations

Ethical approvals were obtained from a Clinical Research Ethics Committee in Hong Kong Joint and an Ethical Research Committee of the research site in Macau, which followed the medical research ethics principles in the Declaration of Helsinki and the compliance of International Conference on Harmonization of technical requirements for the registration of pharmaceuticals for human use- Guideline for Good Clinical Practice. The full description of the study was explained to the participants. Participants could decide to voluntarily participate in the study and terminate their participation at any time without affecting their care. When participants had fully understood their rights and data confidentiality, each one was asked to sign a written consent form. All of the collected data were locked and stored for 6 years in hospital and destroyed after the completion of the aforesaid storage period. This study was registered in the Chinese Clinical Trial Registry which is the representative registry of China to join WHO International Clinical Trial Registry Platform.

## Results

### Description of interview participants

Of 10 participants recruited, 7 were male and 3 were female with an age range from 39 to 77 years (average age of 60.5). Half of them received elementary education and were retired. The majority of participants used iOS smartphones.

### Findings from the interviews

Results from the interviews presented post-PCI patients’ perceptions of using the mCR app. Patients’ experiences of using mCR app were captured by four themes: feasibility of mCR app, benefits from mCR app, advocator for better hospital care, and recommendation for mCR app improvements (Table II).

**Table II.** Contents of themes

Themes	Subthemes	Contents
Feasibility of mCR app	Relevance and practicality Acceptability and convenience Barriers to use	Fruitful and intensive information Multi-functional support Acceptability Convenience Unstable server support Difficult for old-aged user
Benefits from mCR app on well-being	Enhance knowledge of disease management Improving well-being Promoting self-efficacy on healthy lifestyle	Physical Psycho-emotional Exercise Diet Health indicators self-monitoring Medication compliance
Advocator for better hospital care	Extension of hospital service Promoting positive relationship between patients and healthcare providers	Recognition of hospital care Gain more confidence on hospital service
Recommendation for mCR app improvements	Prolong service duration Improving technical backup Consideration of transferability Supports from healthcare system to help this program for long term	Stabilize network support Optimize data entry design To public To relatives To all PCI patients in Macau Financial support Technical support Political support

#### Feasibility of mCR app

The first theme was “feasibility of mCR” which described the participants’ experiences regarding how accessible or usable the mCR app is in their phase II CR after PCI. These criteria included relevance of practicality, acceptability and convenience, and barriers to use.

##### Relevance and practicality

Relevance and practicality were described as whether mCR app provided valuable, necessary and relevant information in an effective approach to support post-PCI patients engaging in the phase II CR after hospital discharge. The majority of the participants (9/10, 90%) mentioned the mCR app gave fruitful and intensive CR education information that was comprehensively related to CR and post-PCI recovery process. Additionally, the evidenced-based mCR app contents were edited and designed by local CR healthcare professionals. Participants appreciated that mCR app efficiently issued reliable and safe information regarding post-PCI recovery.

The mCR app is a very good idea. The information provided is convenient for patients to gain more preliminary understanding about their disease. It includes many videos … the exercise videos support me in learning and doing exercises at home. When I forgot what the doctor or nurse had explained, the app made recalling the educational information convenient. Also, the app helps me record my health condition, such as blood pressure, blood glucose, and body weight. These data are highly related to my health. The app transfers all data to healthcare providers and lets them update and track my health condition. These functions of the mCR app are very useful for post-PCI patients. (C9)

The mCR app is convenient because it contains a lot of functions. There are explanations and guidelines for medication, cardiac rehabilitation process and introduction to appropriate exercise. As you know, this kind of information are widely published in the Internet and I have to choose by myself! The searching process is time consuming, not easy, and it may be difficult for the elderly to do it. When I had the mCR app, I could immediately get the information I wanted because this app was particularly designed for post-PCI care! The information here is reliable and safe as it comes from healthcare professionals. It helps answer the common questions about signs and symptoms of heart disease, self-care notifications after PCI, maintaining physical exercise, healthy diet, and compliance to medication therapy. The education material provided here is very clear and useful. This app is very practical because it provides fruitful education, health data monitoring by nurses, and support communication with nurses. I think mCR app is worthy of promoting to all patients who are discharged to home and receiving CR. (C3)

Multi-functional support of the mCR app assisted involvement of post-PCI patient participating in the phase II CR with better compliance and understanding on the CR goal. Some participants (7/10, 70%) stated that push-up notifications of self-care monitoring (such as BP, pulse, weight, and blood glucose [BG]), medication taking, and exercise recording facilitated their adherence to the CR advice and promoted their achievement of CR treatment goals.

The most practical feature is the reminders. The mCR app is very useful because it reminds me something very important to me, taking medication and check BP. Using the app is very simple. I just click the button to know what I need to do and fill in the number to complete the self-monitoring tasks, such as daily medication taking. After I take my medication, I tick the list in the app to complete my self-care tasks. When I want to know my BP trend, I click the summary to view my BP history. (C5)

The majority of participants (8/10, 80%) expressed that the enquiry feature of mCR app could help them clarify their doubts about self-care of post-PCI after hospital discharge. Their non-emergent enquiries could be answered within 1 or 2 days, but participants commented that this direct communication with healthcare professionals was efficient and effective.

I like the enquiry function because I can communicate via one-to-one questions and answer with a nurse. I try my best to do good self-care because I understand that nurse is monitoring my health condition. If my daily health data was problematic, nurse would text me and remind me. For example, I texted nurse that I felt stomach discomfort after taking medication, and she gave me advice for relieving the discomfort and reminded me the possible side-effects of my medication. She suggested me to see gastroenterologist if her advice does not work. So, the enquiry function can solve my problem, and her answer is very helpful! (C1)

Another way of multi-functional supports of the mCR app was the participants’ weekly performance summary containing their weekly BP, pulse, BG, weight, smoking status, medication compliance, and exercise condition. It was an output summary of their health data input through the mCR app. Participants (8/10, 80%) mentioned that this performance summary facilitated their understanding of their health condition and their CR goal achievements. Furthermore, it was convenient for them to bring and show this summary to doctor when they had their medication appointments.

I appreciate the mCR app because it is very practical and useful. The time for reminding me to input my health data is fit for my daily schedule. It wouldn’t disturb me and it just shows a message to remind me daily. I can review the health data and I know more about my health condition every day. This is a good idea that I can make my record in the app. It’s convenient for me to export a hard copy of my health data and take the record when seeing my doctor in the government hospital. The doctor in the government hospital gave me a logbook to record my BP six months ago, but I lost the Logbook. It doesn’t a matter! I can have my BP record from the app for my governmental medical follow-up now. (C10)

##### Acceptability and convenience

All participants stated they were able to use the mCR app because the contents were in Chinese and had pictures, and the overall functions were easy to use. The mCR app was setup in the smartphone, thus it was convenient to carry, retrieve the educational information, and input health data entry at any time. The majority of the participants (8/10, 80%) mentioned that the health data entry method was simple and completing the self-care monitoring tasks was fast. On the other hands, the push-up notifications for motivating the compliance of self-care were individually scheduled to fit the participants’ life schedules.

The contents of mCR app are easy to understand as the interfaces for information viewing and data input are simple. This app is a Chinese app and it is not difficult to use. In general, this app is easy to use and practical for self-care. It’s really helpful! (C2)

The mCR app was setup in my smartphone, in the front page of my phone, so it is convenient for me to remember. It is like a reminder for me to take my medication and check my BP. If I’m not sure whether I’ve taken my medication, I’ll look up the task completion in the app. The app also shows me information of heart healthy self-care, physical exercise, and food that should be avoided. (C8)

##### Barriers to use

Some participants (4/10, 40%) considered that the mCR app might be difficult for the elderly due to unfamiliarity in using the smartphones, small front size of mCR app, or illiteracy of patients.

Although this mCR app is suitable for me, I think the older people who are illiterate or not familiar with using a smartphone may have difficulty with the mCR app. But, nurse can teach the relatives or the younger family members to use it and how to assist the elderly to use the app. (C6)

#### Benefits from mCR app on well-being

The secondary category represented a number of benefits on well-being in using mCR app to explain the degree of effectiveness of the mCR study. Participants described enhancing CHD disease management knowledge, improving physical performance and psychological comfort, promoting self-efficacy on CV health by lifestyle change via regular exercise and cardiac-healthy diet, and encouraging self-care behaviour in self-monitoring for common CV health indicators and medication adherence, were found in the mCR app using experience from cognitive, physical, psychological, attitudinal, and behavioural changes.

##### Enhance CHD disease management knowledge

The majority of participants (9/10, 90%) stated the educational information provided by the mCR app was comprehensive and helped patients understand more about CHD disease management and self-care knowledge after PCI, including CHD disease knowledge, medication therapy, physical exercise guidance, dietary advice, stress coping and relaxation, and CV risk factor management to facilitate a healthy lifestyle. Moreover, the educational material facilitated easy and effective understanding via text, pictures, and videos.

I think the mCR app gives me systematic and comprehensive information about post-PCI recovery and helps me arouse awareness of self-care after PCI, including physical exercise, diet, self-monitoring of BP, weight, and other living habits. I have no concept about healthy lifestyle before the onset of my heart disease. This app really helps patients gain more knowledge on disease management and let them follow healthy instructions. (C6)

##### Improve physical performance and psychological comfort

Most participants (7/10, 70%) mentioned that the clear exercise instructions provided by the mCR app could assist post-PCI patients in adhering to the CR recommendations of exercise frequency, intensity, and type via videos, and photos at any time.

At the beginning of post-PCI hospital discharge, I felt slight chest discomfort sometimes. I initially didn’t know how to do warm-up exercise, how to keep exercise intensity, and how to perform cool-down exercise and just exercised by my own way. After the training of CR exercise class and following the instruction of exercise in the mCR app, I gained knowledge on doing appropriate exercises and now I follow the exercise guide. Now, I feel I have recovered well. When I exercise, I haven’t felt discomfort and my body is much better than before. (C7)

Many participants (8/10, 80%) implied that the educational information, communication feature, and their health data summary were a patient-oriented approach of post-hospital discharge follow-up, which made them feel heart-warming, relieved, comfortable, and being supported at the end of mCR program. One participant shared his experience:

When I began to use the mCR app, I was quite confused and hesitated because I had to do something seem to be extra … to follow the daily task, to take medication and check BP. I was worried I’d forget to do that. I treated it very seriously as I knew this process was good for me. I think I nearly completed all the tasks. When I viewed my weekly summary of my health data, I felt very good and relieved. I thought this app was with me and I knew the healthcare professionals were taking care of my condition. I was relieved and my worry was reduced. (C6)

After PCI, I was quite anxious and worried. But I had this app … at least I had some information to read and I understood my process of recovery and my health situation. It brought me comfort as I knew what was good for my health. It helped reduce my worry. On the other hand, I felt comfortable when my enquiry was answered. I thought the mCR app was providing certain types of psychological or mental support through information, communication, and the health data record. Thus, I’m willing to use it. (C2)

##### Promote self-efficacy at establishing healthy lifestyle

The educational contents, push-up notification, and health data input from mCR app promoted and supported participants to be confident to follow the advices given by the CR team, with consultations from cardiologists, cardiac APNs, physiotherapist, dietitian or clinical psychologist. Most participants (9/10, 90%) addressed that they were motivated by the mCR app to initiate and maintain the healthy lifestyle in regular exercise, cardiac healthy diet, self-monitoring of cardiac health-related indicators, and good compliance to pharmacological therapy. In terms of cardiac-related exercise and diet, participants noted that:

I think the mCR app taught me to exercise, to do self-monitoring of health indicators and to choose healthy food to eat. As I knew nothing about healthy lifestyle and self-care before the onset of my heart disease, this mCR app was good for me. I followed the instructions in the app, such as reducing unhealthy food and drinking alcohol. I followed the guidance in the app, and I knew I had recovered very well in various aspects. I am more energetic, physically and mentally feeling great! So, I know my health condition is improving and I understand what I should do and eat to promote my health. (C4)

I used the app to see what diet was good for heart disease patients as the recommended food were listed in the app. When I shop for food, I access the app to get ideas. Also, at the beginning of the CR class, I used the app to teach me appropriate exercises. Now, I know what I should do, so I don’t have to access the app every day. But, if I forgot something, I’d use the app again. (C8)

In managing and understanding their health conditions, all participants said they were inspired to be aware of self-monitoring their pulse, weight, and BG for diabetes patients from the educational contents of the mCR app. In addition, the push-up notification and medication taking record in the mCR app encouraged participants to adhere to the prescribed therapy. All participants stated they could maintain their behaviour of regular self-monitoring and medication taking.

I considered the mCR helping me to regulate my lifestyle … or to build up a healthy lifestyle because I worked with the app for 3 months. As I really followed the process and performed self-monitoring recommended by the app, I gained the health data such as BP and I knew my health condition was at a good level. If my health data was unsatisfied, liked my exercise or BP, I would know my target of improvement for next week. I’d do much better and hope to gain a more satisfied level of my health condition. This is a way of encouraging me to achieve my goal for recovery. (C3)

I understood many patients would forget to take their medication. I was used to have my anti-hypertensive drugs in the past and I would sometimes forget or not sure whether I had taken my medication. It was good that I had the mCR app as it reminded me to do so. After I had taken my pills, I ticked the task list in the app. So, it wouldn’t cause any misunderstanding for my compliance on all medication taking. This function was very useful! The reminder alarmed at eight o’clock in the morning. It’s very suitable and fitted for my schedule. I open the app to see my record of health data every morning, like I view the stock. After 3 months of using the app, I have already developed the habit of taking medication. (C10)

#### Advocator for better hospital care

The third theme to emerge from the data was “advocator for better hospital care”. When participants shared their experience about what they gained or felt from using mCR app after hospital discharge, their perceptions of the role of mCR app on post-PCI patient care were disclosed. All participants appreciated and expressed that mCR app produced an extension of hospital care and promoted positive relationship between patient and healthcare providers.

##### Extension of hospital CR care

All participants recognized the mCR app as a part of CR program and an extension of hospital CR care. Participants appreciated the mCR app can assist patients follow the CR recommendation and was actively involved in the patients’ daily of living with CHD in the phase II CR. Half of the participants expressed they were happy to join the study and they appreciated mCR app as a smart and unique care design by the hospital to continue phase II CR in Macau. Participants (6/10, 60%) stated they were impressed by the passion on improving care and gained more confidence from the service provided by this non-government hospital.

I appreciate the idea of mCR app in this hospital and it is really a very good service. Because the healthcare professionals are still taking care of patients who are discharged to home … they are not necessary to do this kind of care … I understand! The care responsibility belongs to patient themselves after hospital discharge. The care delivered through the mCR app seems a voluntary work! I appreciate this passion very much and I give a ‘like’, my appreciation, to this hospital. Hahaha! I know the governmental hospital doesn’t have this service. (C5)

##### Promoting positive relationship between patients and healthcare providers

As the use of mCR app was ongoing in phase II CR, the interaction via the mCR app enquiry feature connected the patients with nurses and the CR team. Half of the participants expressed their heart-felt thanks to the cardiac APNs and the whole CR team when they received warm replies from the mCR app through multi-professional supports.

I thank the CR team supporting the mCR app … and appreciate the CR professionals used their own time and effort to develop this program! To be honest, the cardiac nurse was very thoughtful, and she even concerned the continuity about my medication. She’d review the medication given by the governmental hospital to confirm the continuous supply of my medication. The cardiologist and the physiotherapist were very supportive, and they gave me encouragement. The CR team was very good to support my process of recovery. (C8)

#### Recommendation for mCR app improvement

The last category noted the overall satisfactions for the mCR program, and it was rated satisfactorily by the participants when they shared their experiences of using the mCR app. The participants agreed that mCR app was reliable and effective to help post-PCI patients become compliant to CR recommendations. However, participants offered several recommendations to improve the mCR app as to maximize its benefits.

##### Extension of mCR app care duration

The mCR app was designed to support phase II CR; hence, all educational information was permanently for retrieval. Nevertheless, the health data entry, and health data output summary, and enquiry feature would be terminated at the 3 months after hospital discharge. Some of the patients (3/10, 30%) considered the duration of mCR app to be enough to support their recovery. However, half of the participants commented that they would like to continue using the mCR app, which supporting of health data tracking and communication with healthcare providers. They expressed their desire to extend the mCR care until six months or even one year after hospital discharge.

If all functions of mCR app can be offered … for one year after hospital discharge, it’ll be much better for patient’s self-care as some medications (dual antiplatelet therapy) should be adhered for one year. (C6)

##### Improvement of technical backup

Some participants revealed they found difficulty in downloading information or inputting health data due to limitations of internet support. Some participants (2/10, 20%) suggested optimizing the data input method of mCR app, such as applying a numerical keyboard for health data entry and audio-recording for enquiry contact. Moreover, more than a quarter of participants (3/10, 30%) hoped for faster replies to the enquiries in the mCR app, liked the common feature in social communication apps.

Sometimes, I have difficulty in accessing the app. It may be the problem of network or server and I hope this problem could be solved. I suggest the mCR app can link up with some social communication apps, like WeChat. It’d be more convenient for patients to contact with healthcare professionals by moment-to-moment communication. On the other hands, I think if the app supports numerical keyboard and audio-recording input, it’ll be easier for older people to use as texting is quite inconvenient. (C8)

##### Transferability of mCR app

Participants perceived the mCR app to be divided into two sections: CHD health education section and tracking for health indicators. More than half of the participants (6/10, 60%) suggested that common health educational section was useful and could be shared or transferred by the mCR users to their relatives, friends or other post-PCI patients in Macau. Moreover, the CHD education would be open on the hospital website to the public, CHD and post-PCI patients as to arouse their awareness of CHD prevention and management. However, for the sections that included tracking personal health indicators and one-on-one communication, participants agreed to maintain the current design because it involved confidentiality.

I recommend this mCR app to be separated into two parts. One is for educational knowledge which means the information related to common CHD knowledge, prevention and healthy lifestyle recommendation in exercise and diet. This kind of health education can be open to public who do not have heart disease and let them know more about CHD prevention. I hope to freely share the information with others who are interested in. Another part is for my personal health data tracking. This part should be protected and only offered to patients. If the mCR app makes such changes, it’ll help many people. (C2)

##### Supports from healthcare system to help this program for long term

All participants agreed and hoped that the mCR program could be promoted and applied in the routine phase II CR for post-PCI patients in the future. To support the program in the long run, all participants suggested the local healthcare system and Macau government to provide financial, technical, and political support for the long-term development of the program.

I think the mCR app can be freely offered to patients receiving medical subsidy from Macau government. It is not difficult for government to support this app for service nowadays. (C1)

I think hospital may not have sufficient resource to support the mCR app in a long-term development. If government is able to provide support, such a financial and manpower support, some technical supports, such as wearable devices for exercise or wireless BP monitor, will be considerable to apply and link up with this app. This concept can be promoted to keep tracking on patient’s health condition after hospital discharge in future. (C9)

## Discussion

This study was one of the early studies to investigate an mHealth application on phase II CR among Chinese post-PCI patients, particularly for the older CHD patients. The findings of this evaluation revealed that the mCR app was feasible for post-PCI patients in their phase II CR recovery. These are referred to the data for the sub-theme of “relevance and practicality”. It was confirmed by the users’ recognitions which reflecting fruitful and intensive CR education related to disease recovery were provided via the mCR app. These results were different with pervious findings that older adults passively used mobile phones due to fear or hesitation of using unfamiliar and advanced technology (Kurniawan, 2008). Other sub-themes of findings in “acceptability and convenient”, however, expanded the understanding about simple to operate with Chinese app contents, supported with photos and videos. mCR users expressed that mCR app delivered with push-up notifications, one-on-one text messages for enquiry and weekly health status summary were user-friendly and convenient.

CR engagement, physical and psychological benefits achieved during the mCR study were presented. These were great concerns for the participants that the mCR app can contribute to health and well-being by promoting CHD and CR knowledge, physical performance and psychological comfort, self-efficacy on CV risk management, and behavioural change. The psychological distress, such as anxiety and depression, were commonly found among Chinese patients undergoing cardiac catheterization (Chair et al., 2013; Taylor & Molassiotios, 2001). More depressive symptoms contributed a lower self-efficacy which decreasing in physical activities (Siow et al., 2018). The evaluation disclosed that psychological distress of the mCR participants would be relieved with the use of mCR app. Participants commented that they felt heart-warming, relieved, comfortable and being supported in the mCR program via its features including educational information, enquiry features, and health data log review. In addition, the educational information related to exercise encouraging the participants to adhere to CR exercise recommendations and motivating them to exercise. This finding supported the result achieved from a prior mCR experimental study (Pfeaffli et al., 2015). For physical performance improvement, the finding was among the pervious mHealth CR studies recording a favourable intervention effects on walking distance improvement and sitting time reduction (Piotrowicz et al., 2014; Varnfield et al., 2014; Worringham et al., 2011).

The effects on promoting self-efficacy on healthy lifestyle regarding exercise and diet were confirmed. The majority of participants expressed their confidence and ability to initiate healthy exercise and diet because of the motivation by the educational support, push-up notification, health data feedback and text message communication from the mCR app. Also, the mCR participants noted that they were motivated and felt empowered to maintain self-monitoring and good adherence on medication therapy. These self-management behaviour changes were integral to optimize CV risk factor control (such as BP and lipid control) and prevent the progression of CHD-associated complications (Fihn et al., 2012). Finding of self-efficacy improvement was inferred to provide some support for the mobile phone-based CR intervention manipulating key components of SCT. This indicated some insights into the conceivable mechanism of the mCR intervention on self-efficacy in cardiac exercise and diets which improved the patients’ ability to make a healthy behavioural change (Fihn et al., 2012; Maddison et al., 2015). The effects of such interactive patient supporting aid encouraged self-regulation of important health behaviour change and variables relevant to CV health as well as maximizing the improvement of health-related quality of life (HRQL) (Johnston et al., 2016). Another reason supporting the positive findings of improving HRQL might include the improvements of self-efficacy in cardiac exercise and diets which promoting the patients’ ability to make healthy behavioural change (Fihn et al., 2012).

mCR app users recognized the mCR app as an advocator of better hospital care and they suggested some recommendations for mCR app improvements. The majority of participants expressed they appreciated and impressed the passion of mCR app on improving the advanced hospital care. Another key aspect of the intervention within mCR app was to facilitate communication between the patients and the CR team. These findings from evaluation were similar to a mHealth hypertension management program that patients felt empowered from engaging in their disease-related self-care (Grant et al., 2019). Some concerns were raised over the difficulty for elderly or illiteracy and which was found in some existing literature (Hallberg et al., 2015; Morrissey et al., 2018). For those patients who were unfamiliar in using the mHealth support, their family caregivers or primary caregivers might be appreciate and were suggested to manipulate the mCR app. To promote better use of mCR app and maximize the benefits of CR which might have led to stronger study outcomes, some participants recommended additional comments for mCR study, including extension of service duration, improvement in technical support, consideration of transferability, and obtaining support from regional healthcare system.

### Strengths and limitations

This study was embedded within a mCR study as to support the integration of research findings and to achieve the overall study objectives. Qualitative approach is ideal for exploring the mechanisms, understanding the experiences and maximizing future dissemination of adoption of the mCR interventions. Content analysis is able to prompt preliminary understanding and exploration of core areas and key features of intervention when concerning implementation in further application (Krippendorff, 2004). The evaluation explored the acceptability of mCR application, understood how the participants’ experiences of using the mCR app in their Phase II CR and gave suggestions of key aspects relating to further implementation to meet patient’s needs. However, purposive sampling was carried out in the earlier stage of mCR study with similar educational level and occupational status, there was under-representation of non-iOS mobile users across the sample due to majority of iOS mobile system among study participants. Therefore, long-term effect of the intervention and integration with advanced mHealth technology are suggested to be investigated future.

## Conclusion

This mCR study was designed for post-PCI patients in Macau to support their self-care and adherence in phase II CR after hospital discharge. To meet the local service gap, the mCR RCT that embedded a qualitative evaluation was conducted. Findings from the qualitative evaluation presented post-PCI patients’ perceptions of using the mCR app. Patients’ experiences of using mCR app were captured by four themes: feasibility of mCR app, benefits from mCR app, advocator for better hospital care, and recommendation for mCR app improvements. The results of mCR program were encouraging and helped post-PCI patients adhere to their CR recommendations. Self-care skills and self-efficacy on managing patients’ life-long illness are intended to improve, which is expected to support their maintenance in healthy lifestyle modification.
